# Challenge in direct Spoligotyping of *Mycobacterium tuberculosis:* a problematic issue in the region with high prevalence of polyclonal infections

**DOI:** 10.1186/s13104-018-3579-z

**Published:** 2018-07-17

**Authors:** Mansour Kargarpour Kamakoli, Sharareh Khanipour, Shima Hadifar, Hasan Ghajavand, Ghazaleh Farmanfarmaei, Abolfazl Fateh, Seyed Davar Siadat, Farzam Vaziri

**Affiliations:** 1 Department of Mycobacteriology and Pulmonary Research, Pasteur Institute of Iran, No. 358, 12th Farvardin Ave, Jomhhoori St, 1316943551 Tehran, Iran; 2 Microbiology Research Center (MRC), Pasteur Institute of Iran, Tehran, Iran

**Keywords:** Direct Spoligotyping, Tuberculosis, Polyclonal infection, MANU2 genotype

## Abstract

**Objective:**

Based on our recent studies the prevalence of polyclonal infection in tuberculosis clinical specimens is more than 50% in Tehran, Iran. With this background, Spoligotyping was performed on clinical specimens and their respective cultures, and we examined whether mixed infections interfere with the results or not.

**Results:**

Based on the Spoligotyping pattern, among the fourteen patients, 57.1% had different genotypes in clinical samples and their respective cultures. These discrepant patterns were suggestive of polyclonal infections in clinical samples with possible overlapping Spoligotype patterns. We propose that in societies with high mixed infections (e.g. Iran), direct Spoligotyping on clinical samples can be controversial.

## Introduction

Tuberculosis (TB) is still a major health problem all over the world. An essential tool in clinical microbiology and molecular epidemiology is genotyping of bacterial pathogens [1]. In many studies the researchers have used cultures for genotyping of Mycobacterium strains. But because of the slow growth of the bacteria in the culture medium, genotyping of samples from *M. tuberculosis* culture is also slowly [2]. Mycobacterium cultures can also be false negative, because some strains require more time to grow in culture [3]. Also, the growth of the various Mycobacterium lineages in the culture medium can be influenced by the expression of different genes in the pathways of metabolism of these lineages [4]. Therefore using a quicker method and using clinical samples directly for genotyping can be very crucial [5]. Several PCR-based methods have been used to study Mycobacterium genetic variation. The techniques include Spoligotyping and MIRU-VNTR typing [6]. In very few studies direct Spoligotyping on clinical specimens was performed [3].

One of the major issues with TB is mixed (polyclonal) infections, which is a person’s infection with several different genotypes of Mycobacterium [7]. MIRU-VNTR is the best and most widely used method for identifying mixed infections in TB [8]. Several studies have used MIRU-VNTR to diagnose mixed infections in clinical specimens and this method has been successful in detecting the mixed infections [9–11]. But a recent article suggests that mixed infections in clinical specimens can disrupt direct genotyping and challenge the Spoligotyping technique [3]. Multiple strain infections may cause false Spoligotypes that have been reported previously [5, 12].

The aim of the present study was comparing the direct and indirect Spoligotyping on clinical specimens and their respective cultures, respectively. We also examined whether mixed infections interfere with the results or not.

## Main text

### Methods

#### Sample collection and microscopy

This study was conducted between October 2017 and January 2018. Samples were from patients with definite diagnosis of pulmonary TB. Ethical reviews and informed consent approval were granted by the Ethical Committee of the Pasteur Institute of Iran. The written informed consent was obtained from all patients enrolled in the study. Study samples were included 14 clinical samples (11 smear positive and 3 smear negative). In our study, 12 clinical specimens including sputa and 2 samples were gastric juices. Clinical samples were classified by microscopy according to CDC guidelines [13].

#### Culture

Fourteen clinical specimens were decontaminated by *N-*acetyl-l-cysteine method and inoculated in Lowenstein–Jensen (LJ) medium.

#### DNA extraction from clinical samples and their respective cultures

For DNA extraction, the volume of clinical sample was 1.5 mL; after centrifugation, 500 μL of pellet was used. For their respective cultures single colony was selected and diluted in 500 μL TE buffer. After that, genomic DNA was extracted from clinical samples and their cultures with Proba-NK DNA extraction kit (DNA-Technology Company, Moscow, Russia) according to the manufacturer’s instructions. The DNA was stored at − 20 °C until used for molecular studies.

#### Spoligotyping on clinical samples and their respective cultures

Spoligotyping was performed in duplicate with a commercially available kit (Mapmygenome Genomics company, India) [14]. The PCR was performed on 10 ng purified chromosomal Mycobacterial DNA and 20 ng DNA extracts from clinical samples. *M. tuberculosis* H37Rv and *M. bovis* BCG (Pasteur strain) were used as control strains. Data analysis was performed by MIRU-VNTR plus [15, 16].

### Results

Based on the Spoligotyping pattern, among the fourteen patients, 57.1% (8/14) had different genotypes on clinical samples and their respective cultures. Direct and indirect Spoligotyping yielded the patterns in 100% (14/14) of the specimens and their respective cultures. Microscopy was negative in 3 sediments after decontamination. Of these, all of them was successful in direct Spoligotyping. In our study, 42.86% at Spoligotype had similarity between clinical samples and culture. According to SpolDB4 Lineage, among the 14 clinical samples, five (35.7%) belonged to MANU2 genotype which had the highest proportion in the genotype of clinical specimens. In clinical samples, other strains were classified into families included three strains (21.42%) from the H4 family, two (14.29%) from the CAS1-DELHI, one (7.14%) from the T1 family and three (3/14; 21.4%) strains were Unique.

On the other hand, among their respective cultures, T1 was the largest lineage (5/14; 35.7%) and other strains were classified into families included four strains (28.57%) from the CAS1-DELHI family, three (21.4%) from the H4 family, one (7.14%) from the CAS2 family and one (7.14%) strain was Unique. Interestingly, 62.5% (5/8) and 37.5% (3/8) of the samples with different genotypes in clinical samples and their respective cultures showed a genotype of MANU2 and Unique strains in their clinical sample, respectively. Additionally, 50% (4/8) and 37.5% (3/8) of the samples with different genotypes in clinical samples and their respective cultures showed Lineage 4 (SpolDB4 lineage T1) and Lineage 3 (SpolDB4 lineages CAS1-DELHI and CAS2) in their culture, respectively. All the Spoligotype patterns in clinical samples and their respective cultures from the 14 TB patients is summarized in Table 1.

**Table 1:**
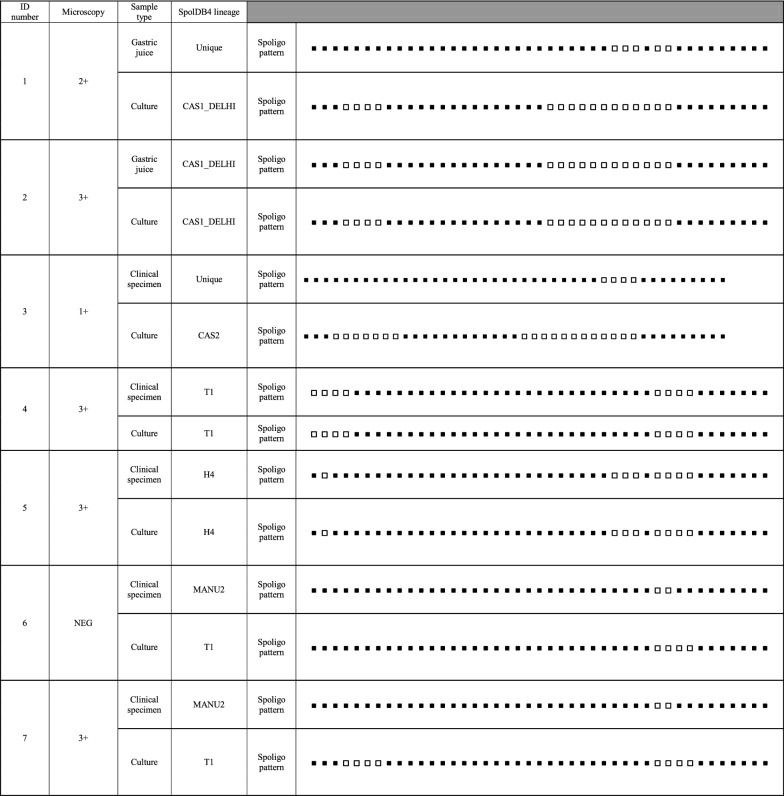

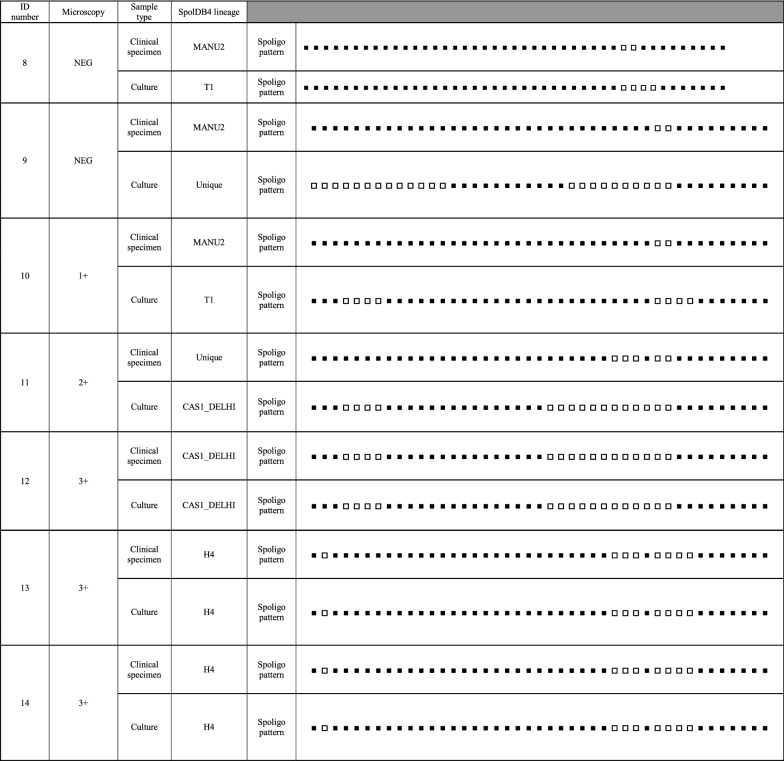
Results of Spoligotyping of the clinical samples and their respective cultures from the 14 TB patients

### Discussion

Our recent studies have shown that the rate of polyclonal infections in clinical specimens is more than 50 percent in our region [9–11]. With this background, Spoligotyping was performed on clinical specimens and their respective cultures, and interesting results were obtained.

The availability of all Spoligotype profiles in our study (100%) is higher than the 98.5% (196/199) found by Sanoussi et al., 90.9% (159/175) by Goyal et al., 49.1% (28/57) by Heyderman et al. on sputa and also higher than Suresh et al. and Zanden et al. on smears [3, 17–20]. These difference may due to DNA extraction methods from sputum [3]. Spoligotyping directly on clinical samples is very effective and time-saving as compared to indirect Spoligotyping on culture. It can also be used for samples that have been cultured negatively or contaminated [21, 22].

Of the 14 studied patients, 57.1% (8/14) had different genotypes on clinical samples and their respective cultures. These 8 samples with discrepant patterns were suggestive of mixed infections in clinical samples with possible overlapping Spoligotype patterns. Similar to our previous results, approximately 50% of mixed infections were observed in clinical specimens [9–11]. In the current study, 62.5% (5/8) of the samples with different genotypes in clinical samples and their respective cultures showed a genotype of MANU2 in their clinical samples. This could be another indication of the presence of mixed infections in these clinical specimens. In this regard, there are several studies that indicate that MANU2 genotype is rare in *M.tb* cultures in Iran. In none of these studies, the genotype MANU2 has been reported [23–30]. In addition, indirect Spoligotyping was performed by our group on 172 *M.tb* isolates, and in none of the strains, genotype MANU2 was observed (Unpublished data). Although two studies have suggested the presence of MANU2 in *M.tb* cultures in Iranian patients, but the percentage of this genotype in these two articles is very small in compare to our study (4.2 and 7.9% versus 35.7%) [31, 32].

On the other hand, Mozafari et al. worked on 1242 clinical samples in Iranian patients and MANU2 genotypes was detected in 25 cases [33]. This may indicate that in direct Spoligotyping, MANU2 is a result of mixed infections in clinical specimens in Iran. Albeit, it was not specific to this region; Lazzarini et al. suggested that MANU2 could be one of the five genotypes derived from mixed infections [34]. Also, the first evidence of the presence of the MANU lineages in Mozambique is the study of Viegas et al. who concluded that some of the MANU2 genotypes could be derived from a mixed infections of Beijing and T1 (or Beijing and T2) [35]. Additionally, in our study 37.5% (3/8) of the samples with different genotypes in clinical samples and their respective cultures showed a genotype of Unique strains in their clinical sample. This may indicate that the mixed infections can also occur in direct Spoligotyping as unique strains.

Our results showed that of 14 TB patients, direct and indirect Spoligotyping were fully successful even for negative smear specimens. These contradict the results of Sanoussi et al. who failed for direct Spoligotyping for their negative smear specimens [3]. Additionally, in a same study 94.4% (135/143) of the Spoligotype levels and 96.5% (138/143) of the lineage level were similar between sputum samples and cultures. They had only two samples containing mixed infections in the sputum samples [3]. On the other hand, in our study, 42.86% at Spoligotype level and 42.86% at the lineage level had similarity between clinical samples and culture. This is due to the large number of mixed infections in sputum samples in our study.

In the current study, 100% of samples that had a different pattern in Spoligotyping between sputum specimens and their culture, contained inter-lineage discrepancies. This may indicate the presence of completely different strains in samples containing mixed infections. In another study, it was shown that MANU2 contains inter-lineage discrepancy of the Beijing and Euro-American strains (T1 and T2 sublineages) [35]. Similarly, in the study of Sanoussi et al. patterns of simultaneous presence of ancestral and modern lineages were observed in the sputum and culture [3].

Additionally, 50% and 37.5% of the samples with different genotypes in clinical samples and their respective cultures showed Lineage 4 (SpolDB4 lineage T1) and Lineage 3 (SpolDB4 lineages CAS1-DELHI and CAS2) in their culture, respectively. In this regard, it has previously been established that the lineage 4 has grown faster (in liquid medium) than the lineage 1 and the ancestral lineage [36]. Also, according to a study by Viegas et al. who considered MANU2 as a mixture of T1 and Beijing, in our study, T1 seems to have more chance of rising in cultures derived from clinical specimens with mixed infections [35].

In conclusion, direct Spoligotyping is very efficient in regions where mixed infections are low in their clinical specimens. On the other hand, in societies with high mixed infections (e.g. Iran) in clinical specimens, we recommend to rule out mixed infection by the MIRU-VNTR method in the first step and after that direct Spoligotyping can be performed more accurately.

## Limitations

The present study must be confirmed by investigation on more clinal samples from TB patients.

## Abbreviations

TB: tuberculosis
Spoligotyping: Spacer-oligotyping
MIRU-VNTR: mycobacterial interspersed repetitive unit-variable number tandem repeat
LJ: Lowenstein–Jensen
